# Toxic epidermal necrolysis in a patient on atorvastatin therapy expressing human leukocyte antigen alleles: A case report

**DOI:** 10.1097/MD.0000000000024392

**Published:** 2021-01-22

**Authors:** Meina Lv, Shaojun Jiang, Jinglan Fu, Yuxin Liu, Siheng Lian, Jinhua Zhang

**Affiliations:** a Department of Pharmacy, Fujian Medical University Union Hospital; b College of Pharmacy, Fujian Medical University, Fuzhou, Fujian; c Department of Pharmacy, Huaihe Hospital of Henan University, Kaifeng, Henan, China.

**Keywords:** ALDEN score, atorvastatin, drug adverse reaction, human leukocyte antigen alleles, toxic epidermal necrolysis

## Abstract

**Rationale:**

Toxic epidermal necrolysis (TEN) is a rare, severe mucosal response of the skin associated with a high mortality rate. TEN is most commonly caused by drugs, and is characterized by extensive skin epidermal exfoliation.

**Patient concerns:**

A 68-year-old woman presented with a rash that had persisted for four days. The patient who had undergone a mitral valve replacement 1 month prior and was taking atorvastatin at the time of admission.

**Diagnoses:**

The patient exhibited more than 30% exfoliation surfaces and the severe drug eruption was considered to be TEN. According to human leukocyte antigen (HLA) allele detection and ALDEN score, HLA alleles which found in this case report may be an cause of TEN induced by atorvastatin.

**Interventions:**

All drugs used prior to admission were discontinued and the patient was given antiallergic drugs.

**Outcomes:**

After 3 weeks following Antiallergic treatment, the rash on patient's calf had subsided, the edema was relieved, and the patient was no longer experiencing pain. After 60 days following discharge, the patient's skin has regrown.

**Lessons:**

This is the first report describing the induction of TEN by atorvastatin in a HLA alleles carrier. For HLA alleles carrier, atorvastatin may need to be used with caution to avoid TEN. Future systematic research is also required to confirm this finding and avoid similar serious skin adverse reactions.

## 1 Introduction

Toxic epidermal necrolysis (TEN) is an acute, life-threatening, severe dermatosis characterized by epidermal loss and multi-site mucositis, and is accompanied by systemic disturbance.^[1]^ It is rare but very serious forms of drug-induced cutaneous adverse reaction. The morbidity of TEN is approximately 2 cases per million individuals each year.^[2]^ The average mortality rate of TEN is over 30%, and the mortality rate of critically ill patients (SCORTEN > 3) is as high as 60% to 90%.^[3]^

Approximately 85% of TEN cases are induced by drugs.^[4]^ The primary sensitizing drugs associated with TEN include antibiotics, anticonvulsants, antiviral drugs, and traditional Chinese medicine.^[3]^ Recent studies have found that the occurrence of TEN is related to the individual human leukocyte antigen (HLA) allele genotype.^[5,6,7]^ The HLA complex consists more than 200 genes on chromosome 6 can be categorized into 3 subgroups: Class I HLA, being recognized by CD8+ T cells, consists of 3 main genes, that is HLA-A, HLA-B, and HLA-C. Class II HLA, being recognized by CD4+ T cells, consists of 6 maingenes, that is, HLA-DPA1, HLA-DPB1, HLA-DQA1, HLADQB1, HLA-DRA, and HLA-DRB1. HLA class I molecules are expressed in almost all the cells and are responsible for presenting peptides to immune cells.^[5]^ Studies have shown that certain drugs that cause TEN are associated with HLA alleles; for example, carbamazepine-induced TEN and the HLA-B∗ 15:02 allele are highly correlated. Moreover, TEN induced by abacavir is correlated with individuals positive for the HLA-B∗57:01 allele.^[5]^

## 2 Case report

**Figure 1:**
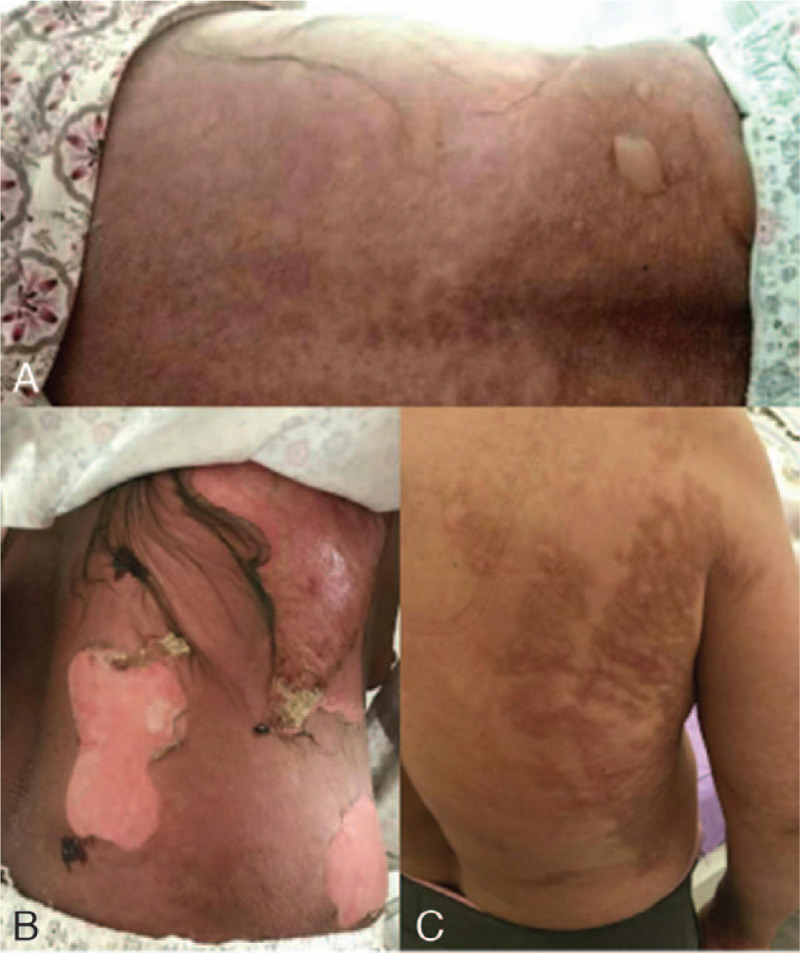
(A) At the time of admission, a burgundy rash was distributed diffusely throughout the patient's entire body. (B) On the fifth day of admission, the patient's facial skin began to peel, and blisters on her trunk began to rupture and peel. (C) After 60 days following discharge, the patient's skin has regrown.

A 68-year-old woman presented with a rash that had persisted for 4 days. She had undergone a mitral valve replacement 1 month prior. Four days before admission, the patient developed a red rash on her face, chest, and back, accompanied by itching and a fever. At the time of admission, she presented with a burgundy rash that was distributed diffusely throughout her entire body. Moreover, some of the lesions were fused together, with a few blisters, itching, tolerability, fever, without skin breakage, subcutaneous nodules, bleeding, and ulcers (Fig. 1A). The laboratory test results revealed: albumin, 34.6 g/L; gamma glutamyl transpeptidase, 45 U/L; glucose, 13.24 mmol/L; creatine kinase MB subtype, 18.0 U/L; creatine kinase, 20 U/L; lactic dehydrogenase, 398 U/L; sodium, 135.9 mmol/L; leukocyte count, 5.19∗10^9^/L; hemoglobin, 98.0 g/L; platelet count, 267 g/L; procalcitonin, 0.130 ng/mL; and fibrinogen, 4.35 g/L. The patient was taking the following medications at the time of admission: 0.125 mg qd po digoxin; 20 mg bid po furosemide; 1 g bid po potassium chloride; 0.625 mg qn po warfarin; and 20 mg qn po atorvastatin.

**Figure 2:**
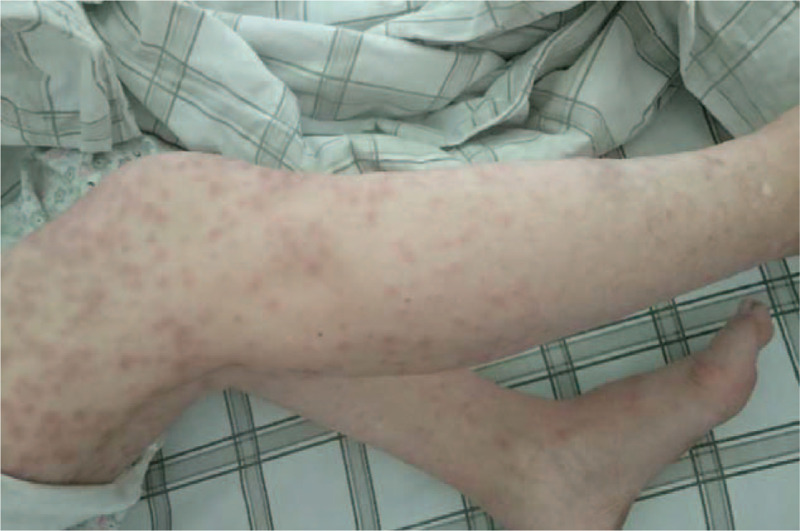
On Day 11 of admission, the patient developed a new red patchy rash on both lower extremities, which was accompanied by mild edema, and moderate blister formation near the thigh.

**Figure 3:**
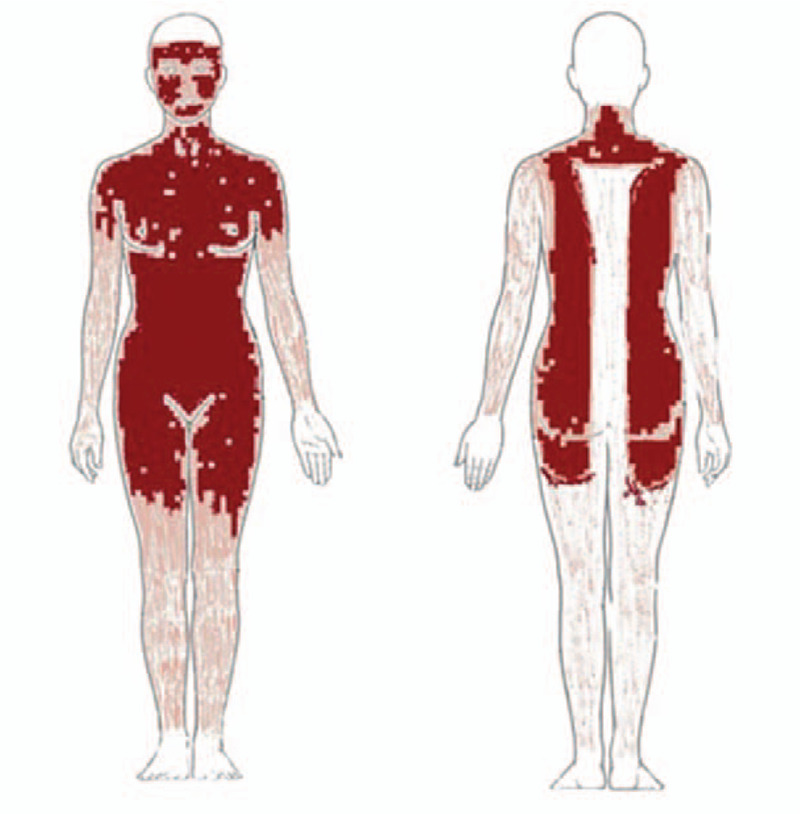
The location and exfoliation site of the rash were observed during hospitalization.

On Day 1 post-admission, all drugs used prior to admission were discontinued and the patient was given antiallergic drugs, including 10 mg qd po loratadine, 10 mg qd po cetirizine, 100 mg tid po vitamin C, 120 mg q12 h po methylprednisolone, 40 mg q12 h ivgtt omeprazole acid, calamine and ethacridine for external use. The following day, the patient developed a large number of blisters that began to rupture over a large area of skin, and was accompanied by a fever. On Day 4, human immunoglobulin was intravenously administered. On Day 5, the patient's facial skin began to peel, and blisters on her trunk began to rupture and peel, which was associated with obvious pain. Mupirocin ointment was applied externally as treatment (Fig. 1B). On Day 11, the patient developed a new red patchy rash on both of her lower extremities accompanied by mild edema, as well as moderate blister formation near the thigh (Fig. 2). On Day 17, after developing erythema, blisters, rupture and peeling, the face and trunk skin were basically healed. On Day 22, the rash on her calf had subsided, the edema was relieved, and she was no longer experiencing pain. Then he was discharged from hospital. After 60 days following discharge, the patient's skin has regrown (Fig. 1C). During hospitalization, the location and exfoliation site of the rash were observed (Fig. 3); since the patient exhibited more than 30% exfoliation surfaces, the severe drug eruption was considered to be TEN rather than Stevens-Johnson Syndrome (SJS).

## 3 Investigations

Since more than 80% of TEN cases are caused by a drug allergy, timely cessation of the sensitizing drugs is the first step in treatment. Screening for suspected drug allergies relies primarily on medical history and previous reports of adverse drug reactions, however, for patients who have used multiple drugs before the onset of TEN, it is often difficult to identify the sensitizing drug.^[4]^ The 2016 British Adult Stevens-Johnson Syndrome/Toxic Epidermal Necrolysis Symptoms Management Guide recommends using the ALDEN score (drug causality algorithm for epidermal necrolysis) to retrospectively evaluate sensitizing drugs for TEN.^[8]^ The ALDEN score was designed based on the results of the Severe Cutaneous Adverse Reaction case-control study conducted from 1989 to 1993. Therefore, we analyzed all of the drugs used in this patient according to the ALDEN score in order to identify those that induce TEN (Table 1). Atorvastatin had an ALDEN score of 4 (the highest score of all drugs taken; Table 1) and was judged to be “probable” for sensitization. Furthermore, the patient had taken warfarin for venous thrombosis of lower extremities 2 years ago and did not induce TEN. Thus, the patient's TEN was most likely caused by atorvastatin.

**Table 1:** Possible sensitizing drugs ALDEN score.

		Values						
Drugs	the duration of use	Criterion1	Criterion2	Criterion3	Criterion 4	Criterion 5	Final score	Sensitization possibility^∗^
Aspirin	9/15–9/30	2^a^	−3^c^	0^e^	0^g^	−1^i^	−2	Very unlikely
Atorvastatin calcium	9/20–10/16	3^b^	0^d^	0^e^	0^g^	1^j^	4	probable
Cefazolin sodium	9/14–9/15	2^b^	−3^c^	0^e^	0^g^	1^j^	0	unlikely
Compound ammonia barbital	9/15–9/16	2^a^	−3^c^	0^e^	0^g^	0^k^	−1	Very unlikely
Furosemide	9/11–9/15,9/20–9/30, 10/1–10/16	3^b^	0^d^	−2^f^	−2^h^	0^k^	−1	Very unlikely
Isosorbide mononitrate	9/20–10/16	3^b^	0^d^	−2^f^	0^g^	1^j^	2	possible
Nitroglycerin	9/15–9/22	2^a^	−3^c^	0^e^	0^g^	0^k^	−1	Very unlikely
Cefoperazone Sulbactam Sodium	9/16–9/30	3^b^	−3^c^	−2^f^	0^g^	1^j^	−1	Very unlikely
Warfarin	9/20–10/16	3^b^	0^d^	−2^f^	0^g^	1^j^	2	possible
Tramadol	9/20–9/30	3^b^	−3^c^	0^e^	0^g^	0^k^	0	unlikely

### 3.1 HLA allele detection

A 2-mL sample of peripheral venous blood was collected from the patient. Genomic DNA was extracted using a DNA extraction kit (Shanghai Baio Co., Ltd.) in accordance with the manufacturer's protocol. The exons of the HLA-A, -B, and-C loci were sequenced using sanger.^[5]^ The results showed that the patient carried the HLA-A∗02:07, HLA-A∗11:01, HLA-B∗15:02, HLA-B∗40:01, HLA-C∗03:04, and HLA-C∗08:01 alleles.

## 4 Discussion

TEN is a serious skin mucosal disease characterized by large areas of erythema, blisters, epidermal exfoliation, and multi-site mucositis, that is often accompanied by systemic dysfunction.^[1]^ The onset is urgent, progresses rapidly, and is associated with a high mortality rate. Thus, there is an urgent need to identify drugs that induce TEN. By using ALDEN to score all the drugs the patient had used, we were able to determine that atorvastatin was most likely associated with TEN in this patient. There have been 6 reports of statin-induced exfoliative dermatitis in the literature, including TEN, but none of the studies tested for HLA-related genes.^[9,10,11,12,13,14]^ In addition, it is mentioned on the atorvastatin drug label that it may cause TEN in extremely rare cases.^[15]^

Genetic factors are also closely related to the occurrence of SJS/TEN. In this study, the patient's HLA exon was sequenced and found to carry multiple mutant HLA genotypes, that may have the correlation with SJS and TEN. A thorough review of the literature reveals that, among these alleles, HLA-B∗15:02, HLA-A∗02:07 and TEN may have the correlation.^[16,17,18]^ The correlation between other alleles and TEN has not been reported, but their association cannot be ruled out, it need to be further investigated. HLA alleles have been proposed as markers of SJS/TEN. Studies revealed a high prevalence of pharmacogenetic markers of drug-induced SJS/TEN e.g., B∗13:01 for dapsone; B∗15:02 for carbamazepine and oxcarbazepine; B∗58:01, A∗33:03 and C∗03:02 for allopurinol; C∗08:01, C∗14:02 and DRB1∗12:02 for co-trimoxazole.^[18]^ HLA alleles which found in this case report may be an cause of TEN induced by atorvastatin. Previous studies have found that HLA-B∗15:02 is an cause of serious adverse reactions in the skin induced by anti-epileptic drugs.^[16]^ Carbamazepine non-covalently bind to proteins or peptides and are presented by MHC molecules after cellular processing, resulting in the HLA–restricted T cell activation.^[17,19]^ Perhaps the mechanism of atorvastatin-induced TEN could be studied in the light of this study.

Atorvastatin is the most common drug for lipid lowering in patients with dyslipidemia, and the proportion of dyslipidemia in Chinese population is as high as 40.40%.^[20]^ Therefore, the population of atorvastatin is very large, but SJS/TEN is very rare, HLA alleles may be a genetic factor. This is the first report describing the induction of TEN by atorvastatin in a HLA alleles carrier. Future systematic research is required to confirm this finding and avoid similar serious skin adverse reactions.

## Author contributions

**Conceptualization:** Meina Lv, Yuxin Liu, Jinhua Zhang.

**Data curation:** Shaojun Jiang, Jinglan Fu.

**Formal analysis:** Yuxin Liu, Siheng Lian.

**Project administration:** Meina Lv, Jinhua Zhang.

**Writing – original draft:** Meina Lv.

**Writing – review & editing:** Shaojun Jiang, Jinglan Fu, Yuxin Liu, Siheng Lian, Jinhua Zhang.
